# The Cultural and Social Challenge of Promoting the Professional Value of Motherhood and Fatherhood

**DOI:** 10.3389/fnins.2022.853329

**Published:** 2022-08-24

**Authors:** Alessandra Pedrocchi

**Affiliations:** Nearlab, Department of Electronics, Informatics and Bioengineering, Politecnico di Milano, Milan, Italy

**Keywords:** women-employment, parenthood (transition to), STEM, neuroengineering, neurorehabilitation, gender gap, gender balance

Speaking of gender balance in professional fields is a point of honor and maturation of our culture. It is now standard, and we are honored that, in all the most organized contexts, especially in science technology engineering mathematics (STEM) disciplines, there is a strong commitment to gender balance. This commitment is usually and mainly expressed by facilitating women's presence, with roles and perspectives similar to those of male colleagues. Huang et al. (2020) thoroughly analyzed the gender gap in STEM academia. Men still outnumber women 2 to 1 in the scientific workforce and, on average, have more productive careers and accumulate more impact in every STEM discipline and most geographic regions. Interestingly, those authors found that men and women publish a comparable number of papers per year and have an equivalent career-wise impact for the same total number of publications. They highlighted the issue of gender-specific sustainability of publication effort throughout an entire academic career, suggesting significant consequences for institutions and policymakers.

Perhaps today, the time is ripe to take one more step, based precisely on the value of what has been promoted so far. What extra step? The work context fosters the importance of motherhood and fatherhood. Being a mother or a father in professional life has an enormous potential value to professional maturity. It is not a ballast for professional development. The present thoughts are grounded on the experience of being responsible for a laboratory where, in the last 10 years, “we delivered” more than ten newborns!

The choice of motherhood and fatherhood is highly personal. Still, it has an enormous social implication and is influenced by the sociological and cultural contexts.

Why does this choice potentially have a substantial professional value?

Let us start with a few considerations. The motherhood and fatherhood decision signifies psychological maturity, an abundance of love, stability of life, assumption of responsibility, and fecundity. Generating and building are the tasks of adults.

Are these factors not important at work and in the profession?

Does being a parent impact the profession, not only in terms of the erratic search for time and commitment on two fronts? What potentially valuable elements does the parent develop in a professional capacity?

By becoming parents, life becomes relativized, and every aspect of life becomes less absolute. The importance of relations grows, egocentrism diminishes, and a balance of different elements is sought (family and professional ambitions, couple, and kids).

The parent is no longer exclusively focused on herself/himself. A natural aptitude for service and recognition of the need of the other grows.

The organization of time, the value of time, and its density change. The willingness to take responsibility is accepted, the seriousness of the commitment matures, and the ability to manage a team grows; only a good team can face the increasing complexity.

Parenthood develops a propensity to empathize and identify with the other's reasons, proposing a glance of optimistic hope toward the world.

Some researchers have studied the effect of parenthood on an academic career and support these empirical observations quantitatively.

Overall, a temporary productivity reduction is associated with birth. However, in the long run, parenthood might positively impact careers. A large study on the international economist community reported that parenthood experience, especially with more than a single child, positively affects the publication outcomes (Krapf et al., 2017).

This latter consideration suggests a further point to our reflection.

The possible positive repercussion of the experience of being a parent in the profession is not automatic. How can the professional context and society promote this positive development?

The answer, I think, is a climate of profound esteem for being parents and a willingness to give credit and room for the responsibility of each person. The focus is the growth of educational and professional responsibility expressiveness rather than a problem of interlocking time, calculation of hours, and work-life balance. Of course, the availability of services (kindergartens, flexibility of hours, and remote working personalized solutions) is essential.

Some studies in the literature support these experience-based thoughts.

For example, Lutter and Schröde showed that women have lower publication rates than men in sociologists' academic communities, and motherhood increases this gap on average. However, they made an exciting observation on their sample. Mothers awarded before their motherhood of grants or prizes showed an inverted outcome: motherhood amplified their performance. The authors suggested that self-confidence matters in determination and success (Lutter and Schröder, 2019).

A US large randomized controlled trial (RCT), CeMENT, on female economists has proved that mentoring programs improved outcomes, increased the number and quality of publications, and enlarged networking and prospective positions (Ginther et al., 2020; Ginther and Na, 2021).

Mentoring and self-confidence support might play a key role.

A dedicated study of this issue in the neuroengineering society is still lacking. A recent US study on STEM employment showed that 43% of new mothers and 23% of new fathers leave full-time STEM employment after their first child, moving to full-time or part-time non-STEM jobs (Cecha and Blair-Loy, 2019). The authors conclude the need for more well-regarded part-time options and ramp-up policies that allow part-time STEM workers to return full-time. They suggest the need for STEM leaders and employers to confront cultural beliefs and set up guidelines enabling STEM professionals, who are already on staff, to manage their family responsibilities, rather than recruiting and training replacements.

A positive example: The Politecnico di Milano offers a budget to new mothers faculty to ensure they can support their research when they return from maternity leave. Someone is waiting for you when you come back with esteem and help you, so you start again soon and gladly!

The value of motherhood and fatherhood is a real investment, worth much more than the maternity leave period's cost, and telling this is good for our society.

## Author Contributions

The author confirms being the sole contributor of this work and has approved it for publication.

## Funding

This study is supported by the European Union's Horizon 2020 Framework Programme under the Specific GA No. 945539 (Human Brain Project SGA3), the Horizon Programme under the Specific GA No. 101057695 (I3Lung), and the project PR19-RR-P5 – FESleg in collaboration with Centro Protesi Inail.

## Conflict of Interest

AP holds shares in AGADE s.r.l. Milan, Italy.

## Publisher's Note

All claims expressed in this article are solely those of the authors and do not necessarily represent those of their affiliated organizations, or those of the publisher, the editors and the reviewers. Any product that may be evaluated in this article, or claim that may be made by its manufacturer, is not guaranteed or endorsed by the publisher.
